# Your Teeth, You Are in Control: A Process Evaluation of the Implementation of a Cognitive Behavioural Therapy Intervention for Reducing Child Dental Anxiety

**DOI:** 10.1111/cdoe.13025

**Published:** 2025-01-10

**Authors:** Jennifer Kettle, Jenny Porritt, Sarah R. Baker, Helen Rodd, Elizabeth Cross, Zoe Marshman

**Affiliations:** ^1^ School of Clinical Dentistry University of Sheffield Sheffield UK; ^2^ Sheffield Institute of Social Sciences Sheffield Hallam University Sheffield UK

**Keywords:** CFIR, cognitive behavioural therapy intervention, dental anxiety, dental care, paediatric, primary health care, process evaluation, qualitative, randomised controlled trial

## Abstract

**Aim:**

To explore the views of patients, caregivers, and dental professionals on the factors that influence implementation, processes, and effectiveness of a guided self‐help cognitive behavioural therapy (CBT) intervention, ‘Your teeth, you are in control’ (YTYAIC), in the CALM trial.

**Methods:**

Semi‐structured interviews were conducted as part of this qualitative component of the process evaluation, and data were analysed using a framework approach based on the Consolidated Framework for Implementation Research (CFIR) and the Five Areas Model of CBT.

**Results:**

Thirty‐seven participants were recruited. Potential mechanisms of action were identified using the Five Areas Model of CBT. Participants felt the intervention may exert change through targeting unhelpful thoughts and feelings (e.g., building trust and perceptions of control) and behaviours (e.g., encouraging effective communication and coping strategies) and facilitating a more positive situational context (e.g., developing more supportive relationships). Enablers (e.g., adaptability, design and delivery) and barriers (e.g., time/resource constraints, cost) to implementation were identified using the CFIR.

**Conclusions:**

This study revealed multiple potential mechanisms of action which could reduce dental anxiety and examined how implementation and contextual factors may influence this change process. The results of the research revealed that the intervention could be implemented in primary dental care and identified the potential barriers which should be addressed to aid successful implementation of the intervention in real world contexts.

**Trial Registration:**

This clinical trial has been registered with an international registry and has been allocated an International Standard Randomised Controlled Trial Number (ISRCTN27579420)

## 1 Introduction

Dental anxiety (DA) is a common problem affecting 13% of adolescents globally [1]. Children's DA is a risk factor for dental caries, which is a major public health problem, and often continues into adulthood if not addressed [2, 3, 4]. When children present with DA in primary dental care, they may require referral to specialist dental services for pharmacological interventions, but this does not address anxiety [5, 6]. Referral of these dentally anxious children may result in them having to wait longer for treatment, prolonging symptoms and travelling further for dental treatment which creates additional potential barriers to dental care and can exacerbate existing healthcare inequalities, as children referred to specialist services for DA and behaviour management are more likely to be from lower socioeconomic backgrounds [7], [8, 9]. Treating children with DA in primary dental care can be time‐consuming, stressful and not well remunerated [4]. Dental professionals (DPs) working in primary care express less confidence in being able to treat children with DA than those working in more specialised services [7]. An effective resource for managing child DA that can be delivered in primary care settings has potential benefits for patients and DPs.

### 1.1 The CALM Trial

CALM is a four‐year (2021–2025) multi‐centre, randomised controlled trial (RCT) that seeks to evaluate the clinical and cost‐effectiveness of a guided self‐help CBT intervention (Your teeth you are in control, YTYAIC) to reduce DA in children attending primary dental care sites across the UK, compared to usual care [10]. CALM will also investigate the effect of the intervention on health‐related quality of life (HRQoL), OHRQoL, referral to secondary care and dental appointment attendance. A process evaluation is being conducted alongside the main trial to consider how the intervention was implemented, the mechanisms of action and the context in which the trial took place, according to the Medical Research Council (MRC) guidance [11]. CALM aimed to recruit approximately 600 children across at least 30 primary dental sites in five UK regions [10]. The inclusion criteria for patient participants in CALM include the following: between 8 and 16 years of age, self‐reporting DA, requiring a course of treatment, and not requiring urgent dental treatment. The comparator in the CALM trial is usual care for dentally anxious patients, typically comprising basic Behavioural Management Techniques as outlined in national and international guidelines [12, 13, 14].

### 1.2 The ‘Your teeth, you are in control’ (YTYAIC) Intervention

The YTYAIC intervention under evaluation is a guided self‐help CBT resource for children, delivered by a DP and accompanying resources for parents and DPs. The YTYAIC intervention was developed based on the Five Areas Model of CBT [15] to target unhelpful behaviours, feelings, symptoms, thoughts, and situational influences which maintain dental anxiety, using evidence‐based psychological techniques to reduce DA in children, including a Message to Dentist communication tool, which provides a worksheet for children to complete (with support from their dental professional) and communicate their worries, what they do and do not want to happen, and what coping strategies and stop signals they would like to use [16, 17]. This activity was seen as beneficial through influencing the child's thoughts, feelings, behaviours, and situational influences that can all operate to maintain a child's dental anxiety (see the Five Areas Model of Dental Anxiety in File S1) [16]. A single‐centre service evaluation found that guided self‐help CBT was feasible to deliver however to date that there has not been a process or outcome evaluation of the use of this intervention in primary dental care [18]. Details of the intervention, mapped to the template for intervention description and replication (TIDierR) checklist [19] and the Five Areas Model, can be found in File S1.

This qualitative study aimed to explore the potential mechanisms of action from the perspective of patients, caregivers, and DPs and examine how YTYAIC was implemented in the context of primary care. The CALM trial is currently ongoing and the quantitative process and outcome evaluations and health economic analysis will be reported on completion of the study.

## 2 Methods

This study is reported following the consolidated criteria for reporting qualitative research (COREQ) guidelines [20] (File S2). Ethical approval was obtained as part of the ethical approval for the CALM trial (East of England—Cambridge South Research Ethics Committee, 11 March 2022, ref. 22/EE/00137).

### 2.1 A Theoretically Informed Approach

#### 2.1.1 Consolidated Framework for Implementation Research

**FIGURE 1:**
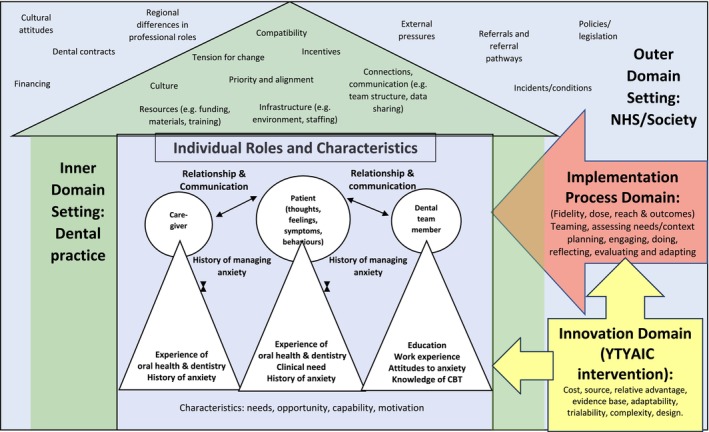
CALM and your teeth you are in control intervention mapped to the Consolidated Framework for implementation research.

Process evaluations should be theoretically informed to rigorously examine the implementation process and aid understanding of why an intervention has or has not worked [21]. This study was guided by the Consolidated Framework for Implementation Research (CFIR) [22]. The CFIR is based on existing implementation frameworks, models, and theories [23, 24, 25] and is intended to explain barriers and facilitators to implementation effectiveness. CFIR consists of 39 constructs across five major domains (that acknowledge the complexity of interventions themselves and how these are implemented in practice, the dynamic interface between inner and outer settings, and the role of various individuals with agency who affect how an intervention works in practice) [20]. The framework has been used effectively in several studies [26, 27, 28] and has been found to capture the complexity of implementation [29]. CFIR was updated based on user feedback in 2022 [30], and this process evaluation uses the updated version as adapted to the CALM trial (File S3). Figure 1 demonstrates how the CFIR can be applied to explore the context of the CALM trial.

### 2.2 Design

Members of the CALM trial youth forum and Patient and Public Involvement and Engagement representatives (PPIE) were involved in the design and interpretation of the process evaluation (see File S2). Interview guides were designed with input from PPIE representative and were based on the CFIR to address the process evaluation questions (e.g., context, implementation, and mechanisms of action) [11].

### 2.3 Research Team and Reflexivity

Interviews were conducted by JK (female), a sociologist with a PhD and experience of qualitative research. Data analysis was supported by ZM, JP, and SB (all female). ZM is a qualified dentist and professor of dental public health, and JP and SB are chartered psychologists; all have experience of qualitative research. ZM, JP, and SB were involved in developing YTYAIC. See File for additional information.

### 2.4 Sampling and Approach

The DPs were purposively sampled by JK and ZM on the basis of gender, region, role/setting (foundation dentist, community dentist, dental associate, practice owner, dental nurse, practice manager), and role within CALM (provider of intervention, provider of usual care or other role). This approach was used to ensure diverse and meaningful representation of views were explored [31]. Twenty‐four potential DP interviewees were contacted by email by ZM in the first instance. Those who responded were contacted by JK and sent information. Four did not respond. Two others did respond but it was not possible to subsequently arrange interviews due to work commitments.

All caregivers of children randomised to receive the intervention who had completed treatment and indicated that they were willing to be contacted about a qualitative interview within the CALM trial were contacted by post and sent an information pack. Those who responded within the timeframe of the study were contacted by JK, and interviews were arranged.

### 2.5 Data Collection

Interviews were arranged with the 18 DPs, ten caregivers, and nine patients who expressed interest. All participants received information sheets prior to their interview. The DPs and caregivers provided written informed consent, and children provided written informed assent. Interviews were carried out by online video call or telephone. The length of interviews ranged from 15 to 85 min with an average length of 53 min. This included 11 interviews conducted as dyads. All other interviews were one‐to‐one with no other people present. All interviews were audio recorded and transcribed verbatim by an external company (Dictate2Us) and checked for accuracy by the interviewer.

### 2.6 Data Analysis

A framework approach [32, 33] was used for analysis using the adapted CFIR and the Five Areas Model of CBT [15, 30]. Data were deductively coded using the analytic frameworks specified and categorised by JK using Microsoft Excel (File S3). Coding and the mapping of data was led by JK and discussed and finalised with the process evaluation team (ZM, JP and SB) during regular process evaluation team meetings. The choice of analytic frameworks, and a credibility check of the mapping and interpretation of the data [34], was discussed during process evaluation meetings with PPIE representatives.

A logic model was developed during the design stage of the CALM trial (see File S4). However, it is important to examine how processes of change operated within the trial as part of the process evaluation. Interviews asked about aspects of YTYAIC that participants felt were most effective and explored participants' perspectives on why these activities had been beneficial and reduced their (or their patient's/child's) dental anxiety. This data was then mapped against the Five Areas Model of CBT [15] to identify mechanisms of action from the perspectives of participants (Table 1). The CFIR was then used as a framework to examine how context may influence these change processes.

**TABLE 1:** Mechanisms of action and contextual influences of YTYAIC mapped to the Five Areas Model of CBT and CFIR.

Intervention component aimed at decreasing children's dental anxiety	Mechanism(s) of change proposed based on Five Areas Model of CBT	Data/evidence (example(s))	Contextual influences (CFIR)
Provides information about how dental anxiety is common (‘Not everyone loves their dentist’ section)	Targeting altered thoughts (e.g., normalisation) and feelings	‘So saying things like “this guide has been created by health professionals and children”, I think is always very well received because it sort of brings in a level into sort of understanding, well, you know, there are other people out there who are worried about their treatment like me’. (S17, principal dentist, male, intervention) ‘It made me feel like I wasn't alone and I wasn't the only one who had fears at the dentist.’ (C05)	
Explains the common unhelpful thoughts that children with dental anxiety experience (using data provided by children in qualitative interviews)	Targeting altered thoughts (e.g., catastrophizing, normalising)	‘So, having a list of common thoughts, especially when you introduce it as “this is what other children have said to us, this isn't us writing a bunch of things, this is what your peers have told us, this is what they think”, makes it more socially acceptable to a child, to then say at the next appointment, “yeah, like these—like I do, I agree with that, I think that too.” (S01, foundation dentist, female, intervention)	
Contains procedural and treatment information (e.g., “The facts” section)	Targeting altered thoughts (e.g., increased understanding, increased self‐efficacy)	‘I think actually having that explanation was really good. Just sort of like, this is what happened, this is what's happening, this is what the sounds mean, this is… that kind of stuff, that was really useful because it sort of gives them a better understanding of what they're going to experience at the appointment.’ (P05)	
Encourages patients and dentists to develop a shared treatment plan (e.g., “Now make a plan” section).	Targeting altered behaviours and situational influences (e.g., promoting effective teamworking)	‘The other thing I really like is the plan for next time. Because I really like that. Again, it reminds me to put a plan into the child's mind to say, well, actually, we're not going to do something too different next time. You've come across most of the things today.’ (S11, community dentist, female, intervention)	
Provides information on cognitive techniques/tools that children can use when at the dental clinic (e.g., “Here's how to take control” section)	Targeting altered behaviours (e.g., promoting effective coping strategies) and thoughts (e.g., positive distraction)	‘And it can prompt them to do things that will help themselves. So, since I've been doing it, lots of children will come with like a fidget toy or headphones or both.’ (S02, associate dentist, female, intervention) ‘But a lot of them have been ticking stuff that they could do while they're in the practice, like maths in their head and things like that.’ (S18, foundation dentist, female, intervention) ‘Maybe, like, distracting my mind with, like, mind games and stuff.’ (C05)	Importance of implementation process. In some of the patient/caregiver interviews, it appeared that the DP was telling the patient to use a particular tool. This may influence this mechanism of action (may not increase perceptions of control): ‘They said to bring air pods for listening to music.’ (C03, patient aged 12, male) Importance of inner setting. General practices could also make use of particular resources to support YTYAIC, such as offering a choice of what to watch on an overhead TV screen as a potential distraction tool’. However, the waiting lists for community settings contributed to some pressure in terms of available time: ‘I think considering our waiting list at the moment is about 700 patients, it's… you know, any extra time is… you know, basically time taken away from seeing that extra patient on the waiting list.’ (S11, community dentist, female, intervention)
Includes a communication tool that can be used by patients to communicate information about their anxiety to the dentist and agree on a stop signal (“Message to the Dentist” section)	Targets altered behaviours (e.g., communicating worries), thoughts and feelings (e.g., increasing trust and control) and situational factors (e.g., developing patient‐dentist relationship)	‘I think it was really emphasised that she had that power almost and it did feel very relaxed and almost like at every stage it was like, “are you okay? Is this okay? Are you happy to carry on?” So, she did feel quite in control.’ (P02, parent, female) ‘in control of what I was doing, and they would talk me through everything and make sure that if, like, I needed them to stop, they would stop. And that, like, it was I was just in control with what was happening, really’ (C05, patient (aged 15)), male ‘It's the reassurance of knowing what's coming or having an ability to stop the dentist if you don't feel comfortable with something.’ (S11, community dentist, female, intervention) ‘And then you can have a good conversation about how they want the appointment to run, what things might help them’ (S02, associate dentist, female, intervention) ‘I think once you sort of go through it with them and sign it, it makes it more sort of official to them as—they can sort of trust me that I'm going to do it, and they seem to respond better once I've done that kind of thing I would say, it does seem to work well generally.’ (S09, associate dentist, male, intervention) ‘Because it creates this like conversational atmosphere before we go and do anything that they're a bit more apprehensive about.’ (S01, foundation dentist, female, intervention) ‘I find it helpful because like then you know what to focus on, so if they've got a specific anxiety like needles or the drill, you can focus on that bit.’ (S06, dental nurse, female, other)	Importance of implementation process. Some dentists completed the Message to Dentist activity with patients at the end of the appointment but this was viewed by a parent as limiting the potential effectiveness of this aspect of the intervention: ‘Once [C04] had completed it—he [the DP] kind of read it through and signed it […] But that was more towards the end, wasn't it, of the appointment rather than at the beginning. So, where they actually can use that information may not—they're not necessarily getting it in the right place of the treatment, if you see what I mean.’ (P05, parent, female)
Encourages children to reflect on their experiences (e.g., “Now you've finished” section)	Targeting altered thoughts (e.g., reappraisal of anxiety)	‘I think sort of asking them to reflect is useful…[…]. so it's sort of forcing them to reflect and actually acknowledge for themselves that this has helped and this has not been as bad. And I think that's really useful.’ (S17, principal dentist, male, intervention)	
Positive reinforcement (e.g., “Time for a reward” section)	Targeting altered behaviours (e.g., increasing engagement through rewarding attendance/effort) and feelings (e.g., positive emotional outcomes)	‘Sometimes they might think… they might be a bit quiet, but when you get to talking about the reward that it really gets them on board.’ (S02, associate dentist, female, intervention) ‘But the one specifically that I'm think of who did it properly wanted to go to the cinema, and yeah, it was “this is what I'm doing” and everything, and I thought, “they've really gone through it together and they've got it”’ (S13, principal dentist, female, intervention)	Importance of Individual characteristics: The idea of agreeing a reward was not viewed as effective by everyone. There were also questions raised about the appropriateness of a reward, particularly when children had not accepted the agreed treatment or if it didn't fit with caregivers' usual approach: ‘I think sometimes it is a bit awkward with the parents because you don't want to sort of—you don't want to overstep the mark.’ (S17, principal dentist, male, intervention) ‘That wasn't the drive for her to get it done at all I think—it was like a bonus, but it wasn't the motivating factor for her to get in the chair.’ (P02, parent, female)
Guided self‐help approach (general). Requires patients and dentists to work through parts of the resource together	Targets altered behaviours (e.g., structured approach, effective communication) and situational factors (e.g., teamworking, improved patient‐dentist relationship)	‘I think they're more up for the treatment and they might still be a bit anxious but it's more manageable, there's sort of a clear resource to work through instead of just trying to manage their anxiety without knowing where to start.’ (S18, foundation dentist, female, intervention) ‘Children's initial response has been very positive because it's given them something… I think a lot of kids don't necessarily like to make that kind of intense eye contact that you start doing when you're grown‐ups talking to each other. So, the guide gives them something to look at and focus on whilst we're talking about how they're feeling and thinking about things. So, even if there was no content in the guide, I think the children appreciate having something to look at and flick through.’ (S01, foundation dentist, female, intervention) I think having it as a paper format is good because it's there. The kids got it, they can see it. You know, they've written the things with their own handwriting.’ (S17, principal dentist, male, intervention) ‘Yeah, I think having the book and being ready to write it in you get, you tend to get an answer whereas, before they would just say, “oh I'm just scared” or “I'm just scared it's going to hurt”, but because you're waiting there ready to write it down, I think that makes a big difference actually.’ (S12, principal dentist, male, intervention)	Importance of implementation process. Some participants felt there was a benefit from providing the resource to the child and working through this together. However not all DPs used the paper‐based resource which could potentially shape how this aspect of the intervention works: ‘I've just kind of remembered the questions or the parts that I thought were useful and I'd say, “right, let's discuss what you want to happen, let's discuss what you don't want to happen.”’ (S15, community dentist, female, intervention)

## 3 Results

Thirty‐seven patients, caregivers, and DPs were interviewed between March 2023 and September 2024 (see File S5 for details).

### 3.1 Mechanisms of Action for YTYAIC

The aspect of the YTYAIC intervention which was discussed in most depth by participants was the Message to Dentist communication tool [18]. Specifically, this activity was viewed as building trust, improving communication and increasing perceptions of control. In most interviews, few concerns or negative impacts were discussed by participants in relation to any aspect of the intervention; the only exception was a concern raised about children with needle phobia seeing the section ‘having an injection’, particularly if this was not in their treatment plan. There were however mixed opinions about the appropriateness and value of the reward section for some children/families, which was raised in DP and children/parent interviews. There was no discussion of how use (or aspects) of the guide reduced the physical symptoms of children's dental anxiety. The interviews emphasised the importance of how the intervention provides a useful structure for the dental appointment, facilitates the patient and dental team to work together and builds a positive patient‐dentist relationship. The factors that might influence the mechanisms of action and the success of the YTYAIC intervention were also discussed and how the intervention was implemented was seen as particularly influential.

### 3.2 Implementing YTYAIC in Primary Dental Care

Based on the interviews, potential enablers or barriers to the future use of YTYAIC were spread across all five domains from the updated CFIR and the detailed mapping of data—with relevant quotes from participants—can be found in File S6. The barriers and enablers discussed by participants in this process evaluation are summarised below and in Table 2.

**TABLE 2:** Summary of Enablers and Barriers of implementation using the adapted CFIR.

Domain and mapped constructs	Enablers	Barriers
Innovation (YTYAIC)	Source Evidence‐base Relative Advantage Adaptability Trialability Complexity Design	Evidence‐base Relative Advantage Cost
Implementation Process	Teaming Assessing Context Tailoring and Adapting Engaging and Doing Reflecting and Evaluating	Assessing needs
Individuals' Roles & Characteristics (DPs, children and parents)	Need Characteristics (Motivation, Capability, Opportunity)	Need Characteristics (Motivation, Capability, Opportunity)
Outer Setting (NHS and society)	External Pressure	External Pressure
Inner Setting (Dental Practice)	Culture Delivery (Compatibility, Mission Alignment, Partnerships & Connections, Incentive Systems, Available Resources)	Delivery (Available Resources)

### 3.3 Innovation (YTYAIC) Domain

As an innovation, YTYAIC (and the online training) was seen as well‐structured [Design] and easy to use [Complexity] and based on accepted evidence [Evidence Base]. However, the DPs were open to further evidence about the effectiveness of YTYAIC, from both the CALM trial and the personal experience of colleagues [Evidence base and Relative Advantage]. It was felt that the resources which formed part of the YTYAIC intervention could be used in different ways [Adaptability]. For example, the message to dentist could be used in isolation (as a printed sheet, with explanation from the DP), without expecting the patient to read the rest of YTYAIC. The intervention group DPs felt it could be trialled with patients outwith CALM without risking negative outcomes [Trialability]. However, some concerns were raised about purchasing the resource outwith CALM [Cost].

In terms of helping patients to manage their anxiety, it was either reported to be more effective compared to usual care [Relative Advantage] or similarly effective, depending on the individual patient (see Individual Domain).

### 3.4 Implementation Process Domain

The interviews identified differences in the way YTYAIC was implemented between patients, between individual DPs and between different dental settings [Engaging and Doing]. The time involved introducing YTYAIC was mostly 5–10 min. Generally, DPs appeared to briefly skim through YTYAIC and highlight particular pages. When considering the future use of YTYAIC, there was some discussion of how colleagues, particularly nurses and receptionists, could support the delivery and how aspects of YTYAIC can be adapted (e.g., the Message to Dentist can be printed and used separately, tools can be recommended, and the questions from YTYAIC can be asked of other patients) [Tailoring and Adapting].

### 3.5 Individual Domain

The DPs interviewed generally showed a high level of engagement with YTYAIC [Motivation]. The DPs felt that using YTYAIC built on existing skills were part of dentistry but it was felt that some professionals may struggle to use the resource more than others [Capability]. As outlined above, using aspects of YTYAIC is seen to be effective at helping patients to manage their anxious thoughts, feelings, behaviours, and physical symptoms and improve the experience of treatment, which meets a recognised need for patients [Need]. Overall, the interviews indicated the importance of the individual domain and how the motivation and engagement of patients, caregivers, and DPs was fundamental to the success of implementation and positive outcomes [Motivation]. Patients benefit from having time at home and in the dental clinic to reflect on and respond to YTYAIC and complete the Message to Dentist; DPs available time relates to aspects of the inner and outer setting [Opportunity].

### 3.6 Setting (Inner and Outer) Domain

These domains have been linked together here as the role of different contracts and the wider NHS structure influences individual inner settings. Overall, there were examples of participants acknowledging the issues caused by dental anxiety and the benefits of addressing this [Delivery‐Tension for change]. The DP interviews highlighted the relevance of time availability to the ability to manage anxiety and use YTYAIC [Available Resources]. The theme of time availability also relates to external pressure from different contractual arrangements in the outer setting domain [External Pressure]. Participants felt that associates, as compared to foundation dentists, dental therapists, and community dentists, had different opportunities to take time to use YTYAIC [Available Resources].

YTYAIC was seen to fit well across different practice settings, including community dentists that receive referrals from general dental practices [Delivery—Mission Alignment, Partnerships & Connections]. The DPs highlighted where YTYAIC fitted with overall goals to help anxious children and provide a positive experience and environment supportive for children [Culture]. Nevertheless, it was noted that not all colleagues would be interested in trying YTYAIC, so there may be differences in values within individual practices. All interviewed intervention DPs were able to use YTYAIC [Delivery—Compatibility] and saw a potential future use for YTYAIC, which they felt could work in different inner settings, and in the context of the outer setting of the NHS and society more generally.

## 4 Discussion

### 4.1 Summary of Findings

Interviews with patients, caregivers, and DPs highlighted that there are several potential mechanisms of action through which YTYAIC may work to reduce anxiety. As a complex intervention, this study suggests that YTYAIC may exert change through targeting behaviours (effective coping strategies and communication), situational influences (e.g., building supportive relationships), and feelings and thoughts (e.g., normalising anxiety), in line with the Five Areas Model of CBT [15, 16]. The participants interview recognised these different elements and provided additional detail of what specific aspects of the intervention they thought worked well. YTYAIC was seen to provide a useful structure for conversations that could obtain valuable information, which could be used for targeted support. The Message to Dentist communication tool was viewed very positively; however, it is important that the ‘active ingredients’ are understood by implementers to ensure that the way in which this aspect of the intervention may work is not undermined, especially in the light of evidence that implementation varied between settings (e.g., providing children with agency in the decisions being made).

The interviews indicated that there are barriers and facilitators to implementing YTYAIC in primary dental care, both within CALM and in the future. In terms of the intervention itself, facilitators included the design, lack of complexity, and relative advantage compared to usual care. Aspects of the inner and outer setting also acted as facilitators and barriers; for instance, communication between colleagues was seen as a facilitator to future use, while external pressure from UK healthcare funding models was seen as a potential barrier. This is related to problems with reimbursement in the current NHS dental contract. More generally, financial pressures within primary dental care may act as a barrier to individual practices purchasing copies of YTYAIC. During the development and testing phase for YTYAIC, a lack of time was acknowledged as a potential barrier [13], and this was also the case in this study. The DPs interviewed who had used YTYAIC and reported that the time involved was manageable and ‘*worth it*’, even in the context of the funding model. Concerns were focused on the potential attitudes of others (while those who had used YTYAIC all planned to use the resource in some capacity in the future). The characteristics of different individuals (the patient, caregiver, and DP) had the potential to be facilitators or barriers; for example, motivation or lack of motivation to engage with YTYAIC. As in previous research, a perceived lack of motivation and interpersonal skills in other DPs were identified as barriers [13].

Initial development and evaluation of YTYAIC was undertaken in a secondary care setting, where the intervention was delivered solely by specialist paediatric dentists [11]. The generalisability of the findings, to the ‘real world’, was readily acknowledged as questionable, driving the need for the CALM trial. Invaluable insights have been gained from the current work, specific to the context of primary dental care, and many of the barriers and facilitators to implementation resonated closely with those found in a hospital setting. It is important that the enablers and barriers identified in this study are discussed and addressed by any future implementers, in the context of their service, in the design stage of implementation.

### 4.2 Implementation and Evaluation of a Complex Intervention in Primary Dental Care

To date, process evaluations conducted in dentistry have not examined how context may influence both implementation *and* mechanisms of action, as recommended for process evaluations [11]. The strength of this paper is that it illustrates the value of undertaking a theoretically‐informed process evaluation to explore these factors and examine how implementation and mechanisms of action operate in different contexts and how this may influence the outcomes of the intervention. The CFIR can provide a useful framework for understanding relevant factors that are perceived to influence the implementation of an innovation [22, 30]. By using the adapted CFIR, this process evaluation has provided a detailed and comprehensive analysis of potential barriers and enablers to the use of YTYAIC within and outside of CALM, which is essential information when considering how this intervention may be scaled up for use in different contexts.

Qualitative approaches explore how individual and system level factors may influence intervention processes and outcomes and understanding complex contextual influencers within trials [30, 35]. Using a qualitative approach within the process evaluation enabled the examination of how YTYAIC has been implemented within CALM, and the factors perceived to be relevant to its implementation in primary dental care. For example, considering implementation in terms of the role of different individuals has highlighted the importance of motivation of patients, caregivers, and DPs. Paying attention to the innovation and implementation processes have highlighted differences in how YTYAIC is used in practice and how it may be adapted in the future. This process evaluation builds on previous research to examine the factors that may influence the acceptability, effectiveness, and feasibility of YTYAIC across different service contexts [16, 17]. The outcome evaluation component of the CALM trial is still ongoing and further consideration of future implementation should be considered in the light of the findings on the clinical and cost‐effectiveness of the intervention when compared to usual care.

## 5 Limitations

A common problem when undertaking a process evaluation of an intervention within a trial is that the implementation barriers/facilitators may have been influenced by the requirements of the trial itself. For example, adhering to the trial protocol and the administrative burden of the trial may have impacted on motivation levels and perceived time pressures associated with YTYAIC [10]. It is also possible that non‐response bias could have influenced the findings. For instance, it is possible that participants who took part in the interviews had different experiences or perspectives to those who did not choose to be interviewed. However, the purposive sampling framework used to recruit DPs was designed to capture a diverse range of experiences and views from participants who had taken part in the trial. Finally, practically, there were time constraints when interviewing DPs, and using a broad framework such as the CFIR may have contributed to less in‐depth examination of specific issues.

## 6 Conclusions

Interviews with patients, caregivers, and DPs found that YTYAIC could be implemented in primary dental care. However, there are potential barriers and enablers to the future successful use of YTYAIC across all five domains of CFIR, which need to be addressed if the intervention is to be successfully scaled up. The study found that multiple mechanisms of action may combine to contribute to a reduction in DA. It is therefore important that these mechanisms of action are understood by those that deliver the intervention to ensure that any adaptations to the intervention do not result in reducing its effectiveness. Indeed, it has been argued that the functions and processes of the ‘active ingredients’ of an intervention, in real world settings, need to be identified and replicated for interventions to work effectively in different contexts [35, 36]. This work has demonstrated the value that implementation science can bring to dental research and process evaluations [37].

## Conflicts of Interest

The authors declare no conflicts of interest.

## Supporting information


**File S1.** Overview of the CALM intervention according to the Template for Intervention Description and Replication (TIDieR) checklist [27] and the Five Areas Model [23].

**File S2.** Consolidated criteria for reporting qualitative studies (COREQ) for Your Teeth, You Are In Control: A process evaluation of a cognitive behavioural therapy intervention for reducing child dental anxiety.

**File S3.** Adapted Consolidated Framework for Implementation Research code sheet (qualitative data) for YTYAIC and the CALM trial.

**File S4.** Logic model of Your Teeth You Are In Control within the CALM trial.

**File S5.** Characteristics of participants.

**File S6.** Potential enablers and barriers to the successful use of Your Teeth Your Are In Control.

## Data Availability

The data that support the findings of this study are available on request from the corresponding author. The data are not publicly available due to privacy or ethical restrictions.
