# Supplementary material for: Your Teeth, You Are in Control: A Process Evaluation of the Implementation of a Cognitive Behavioural Therapy Intervention for Reducing Child Dental Anxiety
Source: Community Dent Oral Epidemiol. 2025 Jan 10;53(2):224–34. doi: 10.1111/cdoe.13025 (PMC11892546; doi:10.1111/cdoe.13025)

**Supplementary file 1: Overview of the CALM intervention according to the Template for Intervention Description and Replication (TIDieR) checklist [27] and the Five Areas Model [23]**

| 1. Name | An assisted self-help CBT resource (‘Your Teeth you are in Control’) to reduce children’s dental anxiety. |
| --- | --- |
| 1. Why? | The intervention was developed because research evidence supports the use of CBT for the management of dental anxiety. This CBT-based self-help intervention was also developed with children, parents, and dental professionals to maximise the acceptability and feasibility of the intervention. |
| 1. What? (Materials) | The intervention consists of:   - A training package and instruction guide for dental professionals - A CBT-based self -help guide for children - A supporting resource for parents/carers   See below for a detailed breakdown of the specific techniques included in the intervention, based on the Five Areas Model [23]. See <https://www.sheffield.ac.uk/dentalschool/research/person-centred-population/child-dental-anxiety/resources> to view the resources. |
| 1. What? (Procedures) | Dental professionals will work through the activities in the self-help booklet with children in line with the step by step guide |
| 1. Who provided? | All dentists will have completed a two-hour online training programme prior to delivery of the intervention (see below for a breakdown of the specific techniques included in the training). |
| 1. How? | The intervention will be delivered face-to-face. Children will be able to take a paper version of the self-help guide home to read through the information in between appointments. Parents/carers will be provided with a summary sheet to take away. |
| 1. Where? | Dental professionals will deliver the assisted self-help intervention in the primary dental care setting in which they work, during the patient’s scheduled dental appointment. |
| 1. When/How much? | The self-help guide will be used with the child at each dental appointment until the course of dental treatment has been completed. This could take between 2 and 6 appointments and 1 and 4 months. Appointment times will vary based on the patient’s clinical and treatment needs, however, based on pilot/feasibility work it is anticipated dental professionals could spend approximately 10 minutes on the intervention at each appointment. |
| 1. Tailoring | Children may not read all sections or wish to complete all activities within the self-help guide. Data will be collected by the dentist during appointments about how well the child has engaged with the intervention and via qualitative interviews. |
| 1. Modifications | Data will be collected on any modifications that are required as a result of the process or outcome evaluation. |
| 1. How well (planned) | Adherence: This will be assessed by collecting data on effectiveness (cost and clinical) for the outcome evaluation.  Context, implementation and mechanisms of action will be explored within the process evaluation. This information will be collected from eligibility logs, case report forms and via qualitative interviews. |

Five Areas Model of dental anxiety outlining the components that the intervention aims to target - Adapted from Williams and Garland (2002)


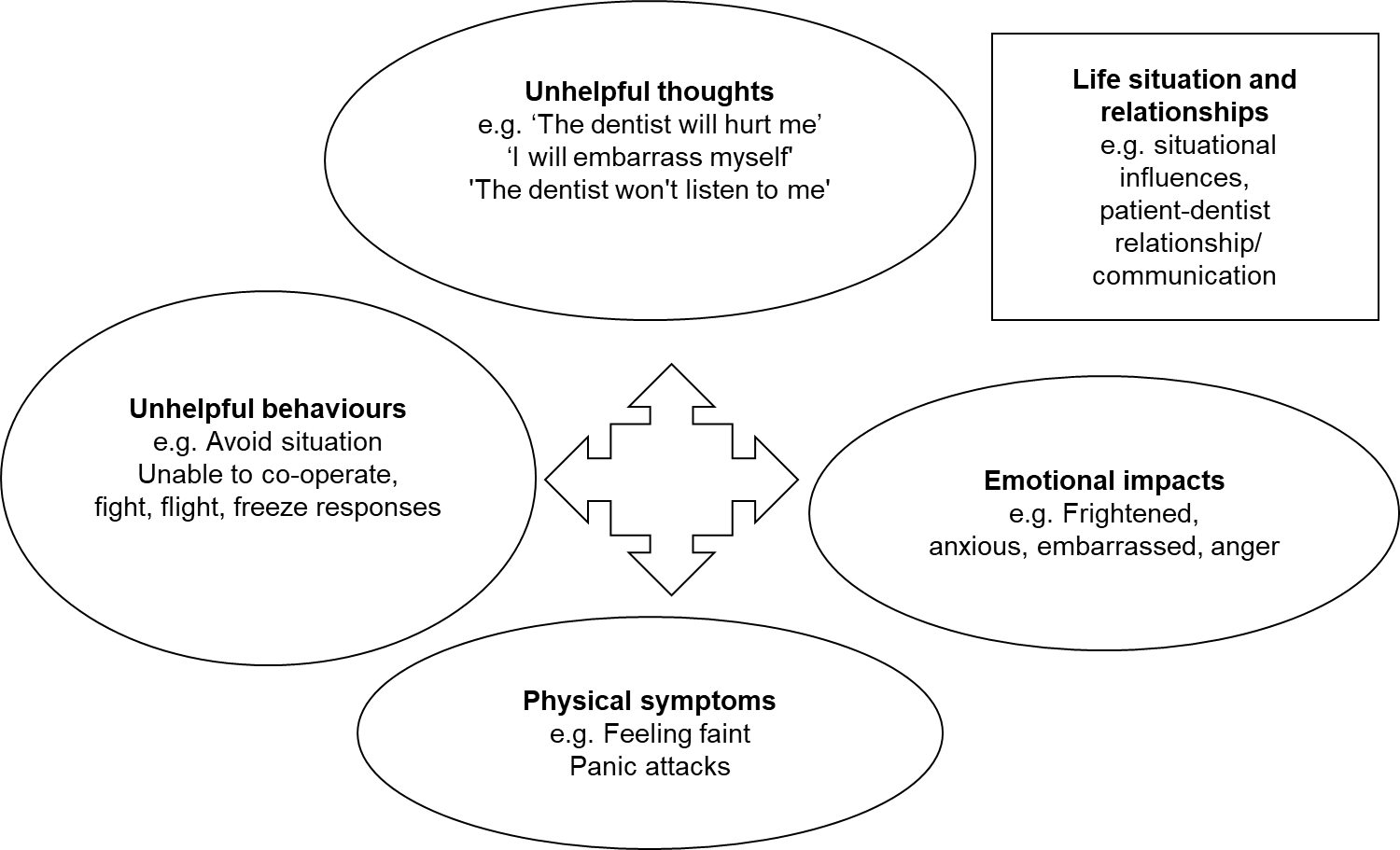

Supplement: Supplementary file 1 — File S1. Overview of the CALM intervention according to the Template for Intervention Description and Replication (TIDieR) checklist [27] and the Five Areas Model [23]. [file CDOE-53-224-s005.docx]
