# Supplementary material for: Your Teeth, You Are in Control: A Process Evaluation of the Implementation of a Cognitive Behavioural Therapy Intervention for Reducing Child Dental Anxiety
Source: Community Dent Oral Epidemiol. 2025 Jan 10;53(2):224–34. doi: 10.1111/cdoe.13025 (PMC11892546; doi:10.1111/cdoe.13025)
Supplement: Supplementary file 2 — File S2. Consolidated criteria for reporting qualitative studies (COREQ) for Your Teeth, You Are In Control: A process evaluation of a cognitive behavioural therapy intervention for reducing child dental anxiety. [file CDOE-53-224-s004.docx]

**Supplementary File 2: Consolidated criteria for reporting qualitative studies (COREQ) for Your Teeth, You Are In Control: A process evaluation of a cognitive behavioural therapy intervention for reducing child dental anxiety**

| **Item** | **Response** | **Included in manuscript** |
| --- | --- | --- |
| Interviewer/facilitator | The first author was the researcher who conducted the interviews. | Yes |
| Credentials | The interviewer has a PhD. Three researchers supported the analysis and all have PhDs (ZM, JP, SRB). | Yes |
| Occupation | The main interviewer is a research associate without clinical training. The researchers who supported the analysis are a qualified dentist/professor of dental public health and two chartered psychologists. The other co-authors who contributed to the paper are a professor of paediatric dentistry and a trial manager. | Yes |
| Gender | The interviewer is female. All co-authors are female. | Yes |
| Experience and training | The interviewer has experience of conducting qualitative research. The other researchers who supported the analysis all of whom also have experience of conducting qualitative research. | Yes |
| Relationship established | The interviewer had previously interviewed one participant for another study. The interviewer had no previous relationships with other participants. | Yes |
| Participant knowledge of the researcher | Details relating to the interviewer’s occupation (see above) were disclosed to participants. | Yes |
| Interviewer characteristics | The interviewers’ aim in conducting the research was disclosed to participants (to explore experiences of taking part in the CALM trial and using the YTYAIC intervention, and to consider how this would work outwith the trial). As someone who is not a psychologist or clinician, the interviewer brought an ‘outsider’ perspective, and explored dental professionals’ responses to anxiety more broadly in order to understand the context in which YTYAIC was used. The interviewer was not able to draw on personal experience of treating anxious patients, using YTYAIC as a dental professional or developing YTYAIC (although insights relating to these aspects were provided by co-authors during analytical discussions). | Yes |
| Methodological orientation and theory | The research was informed by the Consolidated Framework for Implementation Research. | Yes |
| Sampling | Participants were purposively sampled. | Yes |
| Method of approach | Dental professional participants were contacted by email by the PI and caregiver/patient participants were contacted by a letter from the PI. | Yes |
| Sample size | 37 participants were interviewed for the study (18 dental professionals, ten caregivers, nine patients). | Yes |
| Non-participation | Dental professionals, caregivers and patients who were approached and who did not respond, or who did not take part in an interview, are listed in the paper. | Yes |
| Setting of data collection | Data was collected in telephone interviews and in online video interviews. | Yes |
| Presence of non-participants | No non-participants were present during the interviews. | Yes |
| Description of sample | Gender, age (if patient), region, role (if dental professional) and allocation within the trial are included in a table. | Yes |
| Interview guide | Interview guides were designed on the basis of MRC process evaluation guidance and used. Interview guides were reviewed by members of the CALM trial youth forum and Patient and Public Involvement and Engagement representatives rather than being pilot tested. | Yes |
| Repeat interviews | No repeat interviews were carried out. | Yes |
| Audio/visual recording | All interviews were audio recorded. One audio recording failed for a brief period during an interview, and notes from that section of the interview were written up and added to the transcript. | Yes |
| Field notes | Notes were made during the interviews to highlight significant points for further discussion. | Yes |
| Duration | Interviews averaged 53 minutes and ranged from 15 to 85 minutes. | Yes |
| Data saturation |  |  |
| Transcripts returned | Transcripts were not returned to participants for comment. | Yes |
| Number of data coders | Data were coded by the first author only. | Yes |
| Description of the coding tree | Data were coded following the adapted CFIR framework which is provided as supplementary file 3. | Yes |
| Derivation of themes | The process evaluation team met regularly to discuss coding and mapping of data. Data analysis was led by JK. Analytic frameworks were discussed with PPIE representatives. | Yes |
| Software | Microsoft Excel was used to manage the data. | Yes |
| Participant checking | A credibility check of the mapping and interpretation of the data [42], was discussed during process evaluation meetings with Patient and Public Involvement and Engagement representatives. | Yes |
| Quotations presented | Quotations are presented to illustrate findings. Quotations are identified by participant reference. and gender, role (if DP) and allocation within the trial. | Yes |
| Data and findings consistent | The process evaluation team met together and with PPIE representatives to check consistency between the data and the findings/interpretations. | Yes |
| Clarity of major themes | Findings are presented in relation to the five domains of CFIR [30, 38] and Five Areas Model of CBT (mechanisms of change) [23] | Yes |
| Clarity of minor themes | Barriers and enablers to the successful future use of YTYAIC are presented. | Yes |
