# Supplementary material for: Your Teeth, You Are in Control: A Process Evaluation of the Implementation of a Cognitive Behavioural Therapy Intervention for Reducing Child Dental Anxiety
Source: Community Dent Oral Epidemiol. 2025 Jan 10;53(2):224–34. doi: 10.1111/cdoe.13025 (PMC11892546; doi:10.1111/cdoe.13025)
Supplement: Supplementary file 3 — File S3. Adapted Consolidated Framework for Implementation Research code sheet (qualitative data) for YTYAIC and the CALM trial. [file CDOE-53-224-s006.docx]

**Supplementary File 3: Adapted Consolidated Framework for Implementation Research code sheet (qualitative data) for YTYAIC and the CALM trial**

| **Domain and construct** | **General definition** | **Adapted CALM definition** |
| --- | --- | --- |
| **Innovation domain** | **The ‘thing’ being implemented.** | **The ‘Your Teeth You Are In Control’ (YTYAIC) CBT intervention. See TIDIER guidelines for details of the innovation.** |
| Source, evidence base and relative advantage | The group that developed and/or visibly sponsored use of the innovation is reputable, credible, and/or trustable.  The innovation has robust evidence supporting its effectiveness.  The innovation is better than other available innovations or current practice. | The credibility of the YTYAIC intervention and beliefs about its effectiveness and evidence base, compared to other available interventions or current practice. |
| Adaptability & Trialability | The innovation can be modified, tailored, or refined to fit local context or needs.  The innovation can be tested or piloted on a small scale and undone. | How the YTYAIC intervention was (or can be) tailored to meet the needs of the dental clinic and patient group as part of the CALM trial and beyond. |
| Complexity & design | The innovation is complicated, which may be reflected by its scope and/or the nature and number of connections and steps.  The innovation is well designed and packaged, including how it is assembled, bundled, and presented. | The complexity of the YTYAIC intervention (the steps involved) and the quality of its design. |
| Cost | The innovation purchase and operating costs are affordable. | The cost and affordability of the YTYAIC intervention. |

| **Implementation Process Domain** | **The activities and strategies used to implement the innovation** |  |
| --- | --- | --- |
| Teaming | Join together, intentionally coordinating and collaborating on interdependent tasks, to implement the innovation. | How do team capabilities, social relationships, teamwork, and morale influence the intervention? |
| Assessing Needs (e.g. innovation deliverers, recipients) | Collect information about priorities, preferences, and needs of people to guide implementation and delivery of the innovation. | How the needs/priorities of all parties has been met (e.g. for dental team members, work stress, management of DA and young people reduced DA etc.). |
| Assessing Context | Collect information to identify and appraise barriers and facilitators to implementation and delivery of the innovation. | The barriers and facilitators of implementing the resource specific to different contexts (including what can and can’t be changed). |
| Planning & Tailoring Strategies | Identify roles and responsibilities, outline specific steps and milestones, and define goals and measures for implementation success in advance.  Choose and operationalize implementation strategies to address barriers, leverage facilitators, and fit context. | The roles and responsibilities and specific steps which were key to the effective implementation of the resource. How planning was/is important.  The strategies that were used to overcome barriers and adapt to context (or could be used in future). |
| Engaging (e.g. innovation deliverers and recipients) & Doing | Attract and encourage participation in implementation and/or the innovation. | The way in which participation was encouraged and could be encouraged following completion of the trial. Whose support made the implementation of the intervention a success?  The changes which were made throughout the delivery of the intervention/trial. |
| Reflecting & Evaluating  Implementation & innovation | Collect and discuss quantitative and qualitative information about the success of implementation and intervention. | Success of the implementation – what parts/sections did dental team members, young people and parents/carers use and why? What didn’t they use and why? |
| Adapting | Modify the innovation and/or the Inner Setting for optimal fit and integration into work processes. | How the YTYAIC intervention could be refined/modified going forward to fully integrate into primary dental care services.  [Much of what is included here focuses on changes to implementation rather than the innovation] |

| **Individuals’ Roles & Characteristics** | **Roles: applicable to the project**  **Characteristics: the characteristics applicable to the roles in the project based on the COM-B system** | **Roles within dental team and of young people and their carers/parents**  **COM-B factors for healthcare professionals and young people (behaviour: implementation/use of the resource)** |
| --- | --- | --- |
| Role - Leaders (high-level, mid-level, opinion) and implementation facilitators, leads, team members, support and deliverers | Individuals with a high/moderate level of authority or informal influence on attitudes and behaviours, including key decision-makers, executive leaders, or directors, supervisors. Individuals with subject matter expertise who assist, coach, or support implementation, or lead efforts to implement the innovation, or collaborate with and support the Implementation Leads/teams to implement the innovation. | The influence of people with different roles on the implementation of the intervention (e.g. commitment, involvement, and accountability). |
| Role - Innovation Recipients | Individuals who are directly or indirectly receiving the innovation. | The influence of young people and their carers/parents on the use/implementation of the intervention. |
| Characteristics - Need | The individual(s) has deficits related to survival, well-being, or personal fulfilment, which will be addressed by implementation and/or delivery of the innovation. | How do the aims, wishes, and needs of young people, parents/carers and dental team members influence the use of the intervention?  Does the intervention work differently for people based on their needs (e.g. level of DA)? |
| Characteristics - Capability | The individual(s) has interpersonal competence, knowledge, and skills to fulfill role. | Does the psychological capability (e.g. knowledge, understanding) and physical capability (e.g. skills) of young people, parents/carers and dental team members influence the use of the intervention?  Does the intervention work better for people who have certain skills/knowledge/understanding?  Are there individual level capability barriers? |
| Characteristics - Opportunity | The individual(s) has availability, scope, and power to fulfill role. | Do social opportunity (e.g. norms, influence, support) or physical opportunity factors (e.g. time, space, resources) influence the intervention for young people, parents/carers and dental team members (at the individual level)  Does the intervention work better for people who have certain opportunities/resources available? |
| Characteristics -Motivation | The individual(s) is committed to fulfilling role. | Do reflective motivation (e.g. plans, attitudes/beliefs, goals) or automatic motivation factors (e.g. routine, anxiety, emotion) influence the intervention for young people, parents/carers and dental team members (at the individual level)?  Does the intervention work better for people who are motivated to engage or have certain attitudes/beliefs? |

| **Inner Setting Domain** | **The setting in which the innovation is implemented, e.g., hospital, school, city. There may be multiple Inner Settings and/or multiple levels within the Inner Setting, e.g., unit, classroom, team.** | **Dental Clinic** |
| --- | --- | --- |
| General - Structural Characteristics (e.g. physical infrastructure, information technology, work infrastructure) | Infrastructure components support functional performance of the Inner Setting. Examples include layout and configuration of space, data storage, organisation of tasks and responsibilities. | The influence of the dental clinic/service infrastructure (e.g. dental team staffing, storage of resources/patient data, dental environment/room layout etc.) on the intervention. |
| General - Relational Connections & communications | There are high quality formal and informal relationships, networks, teams and information sharing within and across Inner Setting boundaries (e.g. structural, professional). | How the team/structure, communication and information sharing within the dental clinic influences the intervention. |
| General – Culture  (e.g. Human equality, recipient, deliverer and learning centeredness) | There are shared values, beliefs, and norms about the i) inherent equal worth and value of all human beings ii) around caring, supporting, and addressing the needs and welfare of recipients iii) and addressing the needs and welfare of deliverers iv) psychological safety, continual improvement, and using data to inform practice. | How shared values, beliefs and norms about the need to support young people with DA as part of a patient-centred care approach and provide resources and support to dental staff members to help them manage DA influences the intervention. |
| Delivery - Tension for Change | The current situation is intolerable and needs to change. | Is DA a problem which needs to be addressed? |
| Delivery - Compatibility | The innovation fits with workflows, systems, and processes. | Can the intervention be used within the dental clinic context? |
| Delivery - Relative Priority and Mission Alignment | Implementing and delivering the innovation is important compared to other initiatives.  Implementing and delivering the innovation is in line with the overarching commitment, purpose, or goals in the Inner Setting. | Do people think it is an [important] intervention (despite competing priorities/demands) and is it in line with the goals/vision of the dental clinic/service? |
| Delivery - Incentive Systems | Tangible and/or intangible incentives and rewards and/or disincentives and punishments support implementation and delivery of the innovation. | The rewards and positive outcomes of implementing/using the resource. |
| Delivery – Available resources (e.g., funding, space, materials and equipment) and knowledge/information | Resources are available to implement and deliver the innovation.  Guidance and/or training is accessible to implement and deliver the innovation. | Is it possible to implement the intervention in the dental clinic with the resources and guidance/training available? |

| **Outer Setting Domain** | **The setting in which the Inner Setting exists, e.g., hospital system, school district, state. There may be multiple Outer Settings and/or multiple levels within the Outer Setting (e.g., community, system, state).** | **National Health Service & Society** |
| --- | --- | --- |
| Critical Incidents and local attitudes/ conditions | Large-scale and/or unanticipated events disrupt implementation and/or delivery of the innovation.  Sociocultural values/beliefs, economic, environmental, political, and/or technological conditions enable the Outer Setting to support implementation and/or delivery of the innovation. | Any significant (e.g. impact of pandemic) or unexpected external events or political/societal attitudes or external situations which have impacted on the delivery of the YTYAIC intervention. |
| Partnerships, Connections, policies and laws | The Inner Setting is networked with external entities, including referral networks, academic affiliations, and professional organisation networks. | How legislation, regulations, standards, partnerships, referrals/referral pathways, networks or professional organisations/memberships (e.g. BDA, BSPD) have influenced the implementation of the intervention. |
| Financing | Funding from external entities (e.g., grants, reimbursement) is available to implement and/or deliver the innovation. | How funding from the CALM trial and other sources has influenced the use/delivery of the YTYAIC intervention. |
| External Pressure (e.g. societal, market, performance) | External pressures drive implementation and/or delivery of the innovation (e.g. mass media campaigns, competing with peer entities. Quality/benchmarking metrics or service goals). | How the NHS dental contract, and competition in dental services (NHS/private) etc. may influence the use/delivery of YTYAIC intervention. |
