# Supplementary material for: Your Teeth, You Are in Control: A Process Evaluation of the Implementation of a Cognitive Behavioural Therapy Intervention for Reducing Child Dental Anxiety
Source: Community Dent Oral Epidemiol. 2025 Jan 10;53(2):224–34. doi: 10.1111/cdoe.13025 (PMC11892546; doi:10.1111/cdoe.13025)
Supplement: Supplementary file 4 — File S4. Logic model of Your Teeth You Are In Control within the CALM trial. [file CDOE-53-224-s001.docx]

| **Inputs** | **Processes and Activities** | **Outcomes** | **Long term impacts** |
| --- | --- | --- | --- |
| **Dental team:**   - 2-hour training and support resources provided to dental team   **Child and parent/carer:**   - YTYAIC resource provided to child - Support resource provided for parents/carers - Time needed during each appointment to work through planned activities (approx. additional 10 minutes per appointment) | **Initial visit:**   - Dental team introduces YTYAIC to patient and patient reads about dental procedures/ equipment and dental anxiety - Patient reads about benefits of attending dental appointment/ having treatment - Patient reads (and develops knowledge of) effective coping strategies/behaviours - Patient works with parent/carer and dental team to complete ‘Message to Dentist’ and ‘let’s make a plan’   **Subsequent visits:**   - Dental team and patient (with support of parent/carer if possible) review YTYAIC guide from last visit (e.g. review ‘How did it go’?’ section) - Update ‘Let’s make a plan’ and ‘Message to dentist’. - Agree on reward at the end of each appointment. | - Increased understanding from child, parent and dental professional about dental anxiety and how to target factors maintaining child’s anxiety - More effective team working and communication between child, parent/carer and dental team - More effective management of children’s dental anxiety in primary care - Reduction in children’s dental anxiety | - Reduced dental anxiety results in children’s increased ability to complete dental treatment in primary dental care - Reduced referral of children with dental anxiety into secondary services for pharmacological interventions for children with dental anxiety (cost savings and reduced waiting times for children with dental anxiety to receive treatment) - Children are more likely to attend future preventative dental appointments - Improved oral health related quality of life of children with dental anxiety |

**Supplementary file 4: Logic model of Your Teeth You Are In Control within the CALM trial**
