# Supplementary material for: Your Teeth, You Are in Control: A Process Evaluation of the Implementation of a Cognitive Behavioural Therapy Intervention for Reducing Child Dental Anxiety
Source: Community Dent Oral Epidemiol. 2025 Jan 10;53(2):224–34. doi: 10.1111/cdoe.13025 (PMC11892546; doi:10.1111/cdoe.13025)
Supplement: Supplementary file 5 — File S5. Characteristics of participants. [file CDOE-53-224-s002.docx]

**Supplementary File 5: Characteristics of participants**

| **Reference** | **Sex** | **Role (including child age when recruited to CALM)** | **Allocation within CALM** |
| --- | --- | --- | --- |
| S01 | Female | Foundation dentist | Intervention |
| S02 | Female | Associate dentist | Intervention |
| S03 | Female | Foundation dentist | Usual care |
| S04 | Female | Principal dentist | Not allocated within CALM |
| S05 | Female | Dental therapist | Intervention |
| S06 | Female | Dental nurse | Not allocated within CALM |
| S07 | Female | Practice manager | Not allocated within CALM |
| S08 | Male | Principal dentist | Usual care |
| S09 | Male | Associate dentist | Intervention |
| S10 | Female | Community dentist | Usual care |
| S11 | Female | Community dentist | Intervention |
| S12 | Male | Principal dentist | Intervention |
| S13 | Female | Principal dentist | Intervention |
| S14 | Female | Associate dentist | Intervention |
| S15 | Female | Community dentist | Intervention |
| S16 | Female | Practice manager | Not allocated within CALM |
| S17 | Male | Principal dentist | Intervention |
| S18 | Female | Foundation dentist | Intervention |
| P01 | Female | Parent | Intervention |
| C01 | Female | Child (age 10) | Intervention |
| P02 | Female | Parent | Intervention |
| C02 | Female | Child (age 9) | Intervention |
| P03 | Female | Parent | Intervention |
| C03 | Male | Child (age 11) | Intervention |
| P04 | Female | Parent | Intervention |
| C04 | Male | Child (age 9) | Intervention |
| P05 | Female | Parent | Intervention |
| C05 | Male | Child (age 15) | Intervention |
| P06 | Female | Parent | Intervention |
| C06 | Male | Child (age 12) | Intervention |
| P07 | Female | Parent | Intervention |
| C07 | Female | Child (age 13) | Intervention |
| P08 | Female | Parent | Intervention |
| C08 | Female | Child (age 15) | Intervention |
| P09 | Female | Parent | Intervention |
| C09 | Female | Child (age 14) | Intervention |
| P10 | Female | Parent | Intervention |
