# Supplementary material for: Your Teeth, You Are in Control: A Process Evaluation of the Implementation of a Cognitive Behavioural Therapy Intervention for Reducing Child Dental Anxiety
Source: Community Dent Oral Epidemiol. 2025 Jan 10;53(2):224–34. doi: 10.1111/cdoe.13025 (PMC11892546; doi:10.1111/cdoe.13025)
Supplement: Supplementary file 6 — File S6. Potential enablers and barriers to the successful use of Your Teeth Your Are In Control. [file CDOE-53-224-s003.docx]

**Supplementary File 6: Potential enablers and barriers to the successful use of Your Teeth Your Are In Control**

Potential Enablers

| **Innovation Domain** | | | |
| --- | --- | --- | --- |
| **CFIR Construct** | **Definition** | **Potential Enabler** | **Quote(s)** |
| Source | The group that developed and/or visibly sponsored use of the innovation is reputable, credible, and/or trustable. | The team behind CALM are seen as credible due to being experts in this area. Previous experience with named individuals and/or the university may be a moderating factor here. | ‘*I have no experience other than what I've read in the papers and the stuff that* [Name of member of trial team’s] *published and* [Name of member of trial team’s] *published and stuff*.’ (S08, principal dentist, male, usual care) |
| Evidence | The innovation has robust evidence supporting its effectiveness. | Dental professionals accept there is robust evidence on CBT. | ‘*Obviously cognitive behavioural therapy, I think it's a proven thing, isn't it? It’s known to work isn’t it.*’ (S12, principal dentist, male, intervention) |
| Evidence | The innovation has robust evidence supporting its effectiveness. | Dental professionals are open to further evidence about YTYAIC from CALM and from colleagues’ personal experiences. | *‘But I think we’ve found from our own experience that it is working, and our therapist works at the* [Name] *dental hospital as well. And they already use it there, so she's got a lot of experience of how useful it's been there in getting children to accept treatment. So, we do know that it... well, from our experience and her experience, that it can help people'* (S07, practice manager, female, not allocated within CALM) |
| Relative advantage | The innovation is better than other available innovations or current practice | YTYAIC is perceived as more effective than usual care. | *‘But I feel that their success is much better, it’s more effective, and it’s quicker, so. And this is just my experience while we have been traditionally doing quite well without the intervention.*’ (S17, principal dentist, male, intervention)  ‘*I think all of them ultimately would have been successfully acclimatised. But I think one or two of them would have, probably without CALM needed a few extra appointments just to get simpler things done*.’ (S01, foundation dentist, female, intervention) |
| Relative advantage | The innovation is better than other available innovations or current practice | YTYAIC has the advantage of bringing different aspects of care together and provides a structure or framework to organise these. | ‘*I think the guide aims to combine it all and tie it all together to bring a structured approach to managing anxiety. So, it's things that a dentist is so familiar with, but not all of them put together in practice when it actually comes to treating the anxiety*.’ (S11, community dentist, female, intervention) |
| Adaptability | The innovation can be modified, tailored, or refined to fit local context or needs. | YTYAIC can be adapted and used in different ways based on what was acceptable to patients. | ‘*Because I found that they found it too much paperwork, even that one sheet that this, I tend to just kind of use almost for my own trigger points to talk about, but I wouldn't flick through and go through this with them. I think I found it a lot easier when it's one-sided, one paper sheet*.’ (S15, community dentist, female, intervention) |
| Trialability | The innovation can be tested or piloted on a small scale and undone. | Dental professionals perceive they can offer YTYAIC to patients without a negative impact on the patient, even if treatment is not successfully completed. | ‘*And again, I mean, you're not going to get it right every time, but I mean, there's no harm in doing it.*’ (S17, principal dentist, male, intervention) |
| Complexity | The innovation is complicated, which may be reflected by its scope and/or the nature and number of connections and steps. | YTYAIC is perceived by dental professionals, patients and caregivers as easy to use. | ‘*I think the guide itself, everything’s been really easy to follow*’ (S05, dental therapist, female, intervention)  ‘*It was quite easy to read and like understand what it was asking to do*.’ (C01, patient aged 11, female) |
| Design | The innovation is well designed and packaged, including how it is assembled, bundled, and presented. | YTYAIC is perceived as well-designed | ‘*It’s a nice size. It’s not too overwhelming. It’s clear. Very, very clear. It looks good*.’ (S14, associate dentist, female, intervention) |
| Design | The innovation is well designed and packaged, including how it is assembled, bundled, and presented. | The training for using YTYAIC in primary dental care is useful and accessible. | *'I think the underlying training gave me a good understanding of what the underlying principles were’* (S01, foundation dentist, female, intervention) |
| **Implementation Process Domain** | | | |
| **CFIR Construct** | **Definition** | **Potential Enabler** | **Quote(s)** |
| Teaming | Join together, intentionally coordinating and collaborating on interdependent tasks, to implement the innovation. | Other staff in the practice can support the delivery of YTYAIC. This could include supporting a child to complete the Message to Dentist before an initial treatment appointment. | *‘I guess it would help if nurses were involved in it too. And certainly with the reception staff because they’re often the first people that a patient will see and speak to.* […] *I think that would help having everybody else on board to sort of say, “well, we could do it like this”, even if it’s not sort of coming from us.*’ (S09, associate dentist, male, intervention)  *‘Either having a nurse or a receptionist or, you know, just somebody who's not in surgery probably be able to sit down somewhere quiet with them to fill it in, maybe at a check-up appointment so that they've got it before the first treatment appointment.*' (S07, practice manager, female, not allocated within CALM) |
| Assessing Context | Collect information to identify and appraise barriers and facilitators to implementation and delivery of the innovation. | Dental professionals perceive a lack of significant barriers to the use of YTYAIC. | ‘*I can't see many barriers to its use*.’ (S02, associate dentist, female, intervention) |
| Tailoring | Choose and operationalize implementation strategies to address barriers, leverage facilitators, and fit context. | Dental professionals have the ability to address potential barriers (such as patients forgetting their copy of YTYAIC) in different ways. | ‘*I put a note in my clinical notes what they said last time in the book, because the first time when they didn't bring it back, I was like, “oh god, I can't remember what we put”. So, I learned from that and write it down now*.’ (S12, principal dentist, male, intervention) |
| Engaging & Doing | Attract and encourage participation in implementation and/or the innovation. | Dental professionals can engage patients in the idea of YTYAIC when introducing the resource. | *‘So, it's kind of encouraging them to break down the appointment into the little bits and figure out which part of the experience is the bit that's worrying them.*’ (S02, associate dentist, female, intervention) |
| Reflecting & Evaluating | Collect and discuss quantitative and qualitative information about the success of implementation and intervention. | Patients and parents respond positively to the idea of providing a Message to Dentist. | *‘The little form at the back, just to let the child know that they're in control of the whole proceedings, definitely that's been really good as well. The patients have liked that, they've liked to think, “yes, I'm…”, you know, “she is going to listen to me”.’* (S14, associate dentist, female, intervention)  ‘*I think it really helped C01 and I guess it would help younger ones* […] *You know, she’s very nervous, so she wouldn’t have had the open conversation probably without these prompts -- without the written cues almost*.’ (P02, parent, female) |
| Reflecting & Evaluating | Collect and discuss quantitative and qualitative information about the success of implementation and intervention. | Patients are using tools recommended in YTYAIC to manage their anxious thoughts, feelings, behaviours. | ‘*The bit they find more interesting, the fact that they can actually bring in music and toys, and it’s a bit different that they might not have really had before*’ (S18, foundation dentist, female, intervention)  ‘*I think that’s a good idea as well because it gives them a little bit of control and then if they’re listening to music, they don’t have to listen to the drill or if they need a little toy to play with or something to like hold or to fidget with. I think it’s a good idea*.’ (P04, parent, female) |
| Adapting | Modify the innovation and/or the Inner Setting for optimal fit and integration into work processes. | Dental professionals are able to successfully use aspects of YTYAIC with patients outwith CALM. | ‘*I do think there's scope for something similar to work very well with adults, simply because it does take a lot of the stress out of the situation because it's a total change of pace, you know, you’re getting this thing out and pointing out “everyone feels like this”, you know, “it’s really common. And we need to make an agreement about what we can do and what we can't do.”* (S01, foundation dentist, female, intervention) |
| **Individual Domain** | | | |
| **Construct** |  | **Potential enabler** | **Quote** |
| Need | The individual(s) has deficits related to survival, well-being, or personal fulfilment, which will be addressed by implementation and/or delivery of the innovation. | Dental professionals perceive YTYAIC as meeting a need for a resource to help children and young people manage their own anxious thoughts, feelings and behaviours and complete treatment, both within the current treatment plan, and for treatment needed in the future. | *‘You're sort of thinking about what’s going to help you. Then that will help overall, I think, because as they grew up older, they will then know what it is that sort of they are anxious about, what they can do to help, and then they can sort of relay that to any dentist*.’ (S09, associate dentist, male, intervention) |
| Capability | The individual(s) has interpersonal competence, knowledge, and skills to fulfil role. | YTYAIC uses the existing skills of dental professionals (for example, interpersonal skills, the ability to communicate effectively, the ability to build trust). | ‘*I would hope that a lot of the dentists that were using this training would have a lot of the skills or the ideas to build on that*. […] *Communication skills, empathy, wanting to help their patients challenge their thoughts and wanting to have that same shared outcome*.’ (S15, community dentist, female, intervention)  '*I feel like anyone could do it if that’s what you mean. I don’t think you need something prior to have done it other than just to be able to do dentistry. Because if you can do that stuff, all you’re really doing is just managing the patient which we do sort of day in day out anyway. It’s just in a different way to how you might have done*.' (S09, associate dentist, male, intervention) |
| Capability | The individual(s) has interpersonal competence, knowledge, and skills to fulfil role. | Dental professionals develop knowledge and competence from experience of working with anxious children that can be used to deliver YTYAIC. | ‘*So not so much in terms of formal training, but just in terms of pure on the ground experience, I've just seen a lot more kids and a lot more anxiety and a lot wider spectrum*.’ (S01, foundation dentist, female, intervention) |
| Capability | The individual(s) has interpersonal competence, knowledge, and skills to fulfil role. | Patients have the ability to understand and respond to the questions in YTYAIC. | ‘*I’ve not had anyone who’s, you know, they’ve had a barrier in doing it.*' (S17, principal dentist, male, intervention)  ‘*It was easy to think of what to say.*’ (C02, patient aged 10, female) |
| Opportunity | The individual(s) has availability, scope, and power to fulfil role. | Dental professionals have flexibility over how much time they spend with patients, which allows the time to introduce and use YTYAIC. This depends on the constructs Inner Setting – Delivery – Available Resources and Outer Setting – External Pressure. | ‘*I think the fact that I can set my appointment length and have plenty of time to see my patients really impacts the way I can do it because it means I don't feel as though I've got a lot of time pressures and then things build up and it doesn't affect the rest of my day*.’ (S03, foundation dentist, female, usual care) |
| Opportunity | The individual(s) has availability, scope, and power to fulfil role. | Patients have the opportunity to engage with YTYAIC at the dental surgery (e.g. a physical resource, a place to look at YTYAIC or listen to the dental professional talking about it). | ‘*I guess if it was some sort of online thing, I don’t know whether the patient’s going to do that though. I guess when you’ve got them in the surgery, you’ve got them there so they can’t say, “I haven’t got time to do that”.*’ (S16, practice manager, female, not allocated within CALM) |
| Opportunity | The individual(s) has availability, scope, and power to fulfil role. | Patients and caregivers have the time to engage with YTYAIC at home. | *'Yes, so I think it's always useful if they've had time to read it at home'* (S15, community dentist, female, intervention) |
| Motivation | The individual(s) is committed to fulfilling role. | Dental professionals are motivated to use YTYAIC which is seen as ‘worth it’. | *‘Yeah if I felt it was going to help and ultimately it would mean I was going to get the treatment done, then to me that’s five minutes worth it because it means you can do treatment like quicker in the future can’t you? So it's all about like being, if it involves a bit more investing in time now then that's fine.*’ (S05, dental therapist, female, intervention) |
| Motivation | The individual(s) is committed to fulfilling role. | The patient is willing to engage with YTYAIC by reading relevant sections, completing the Message to the Dentist, deciding on the tools they want to use etc. | ‘*So for some patients where they pay attention to the booklet, they’ve brought it, they've read it at home, they've considered it, you know, they're really engaged, they really benefit from it, they feel… you can just tell from the next appointment onwards after they've started using the resource that they feel a lot more empowered and then much more communicative about what they want to do*.’ (S01, foundation dentist, female, intervention)  [Example of engaging with tools] ‘*Having something to like fidget with and maybe like being able to like have a distraction in my head and stuff like that*.’ (C01, patient aged 11, female) |
| Motivation | The individual(s) is committed to fulfilling role. | The caregiver is willing to support the patient to engage with YTYAIC at home. | ‘*I think parental involvement is still a big thing with creating the differences between the children.*’ (S15, community dentist, female, intervention)  ‘*Well, before we filled it in, we just read it all through and spoke about what she thought the answer should be. You know, like just ran through it.*’ (P02, parent, female) |
| Motivation | The individual(s) is committed to fulfilling role. | The patient and caregiver are motivated to bring YTYAIC back for subsequent appointments, and bring anything required to use recommended tools. | ‘*There's lots of really helpful tips there and…like patients are quite keen to bring like stress balls or the fidget toys. They've been really popular and like, you'd see the kid just fiddling away with them*’ (S14, associate dentist, female, intervention) |
| **Inner Setting Domain** | | | |
| **CFIR Construct** | **Definition** | **Potential Enabler** | **Quote(s)** |
| Culture | There are shared values, beliefs, and norms about the i) inherent equal worth and value of all human beings ii) around caring, supporting, and addressing the needs and welfare of recipients iii) and addressing the needs and welfare of deliverers iv) psychological safety, continual improvement, and using data to inform practice. | Dental professionals perceive a fit between YTYAIC and practice culture when the practice is focused on being supportive, patient-centred etc. | ‘*We were already, like we said, quite an understanding or quite a generous practice, I feel like it is quite similar. We was already doing a lot of this before. It's just nice to have that kind of take home like that, you know for them to do at home*.’ (S05, dental therapist, female, intervention) |
| Delivery – Compatibility | The innovation fits with workflows, systems, and processes. | Dental professionals have the ability to use YTYAIC in the context of their dental clinic. | *'I think it, the set up has been good for our practice because it’s kind of flown with the way that we work anyway, which is the dentists do a lot of the check-ups and the hygiene therapists do a lot of the treatment. So it’s, that’s worked. You know, it’s been, it's kind of been malleable to fit in our practice*.' (S16, practice manager, female, not allocated within CALM) |
| Delivery – Mission Alignment | Implementing and delivering the innovation is in line with the overarching commitment, purpose, or goals in the Inner Setting. | YTYAIC is perceived to fit with the overall mission of the setting (particularly within community dentistry). | '*In my opinion, I always try and deal with anxiety. Because if you can deal with it once, generally, they’ll be better the next time. And our aim is to see people for years*.’ (S12, principal dentist, male, intervention) |
| Delivery – Incentive Systems | Tangible and/or intangible incentives and rewards and/or disincentives and punishments support implementation and delivery of the innovation. | Dental professionals find the successful treatment of patients who have previously appeared anxious to be personally rewarding. | *'I actually really enjoy treating children who are anxious because I think the reward element and the satisfaction element of it is so lovely when you can overcome that.*' (S15, community dentist, female, intervention) |
| Delivery – Available Resources | Resources are available to implement and deliver the innovation. | The resource of time and how this is used within the inner setting allows for the introduction and use of YTYAIC. | '*If they come in to their check-up and they need some sort of treatment, and, you know, my feeling is that they are anxious or that cooperation is going to be a problem, then, yeah, I would certainly be doing it at the check-up appointment* [...] *with a view that when they come back next time, they're already prepared*.' (S17, principal dentist, male, intervention) |
| Delivery – Available Resources | Resources are available to implement and deliver the innovation. | Having YTYAIC or photocopied sheets physically available allows patients to work through the resource. | '*I mean, if it was like provided, if there was a booklet that you could just give to patients if you think that they’re anxious, and then they can work through that in their own time*.’ (S18, foundation dentist, female, intervention) |
| **Outer Setting Domain** | | | |
| **CFIR Construct** | **Definition** | **Potential Enabler** | **Quote(s)** |
| External Pressure | External pressures drive implementation and/or delivery of the innovation (e.g. mass media campaigns, competing with peer entities. Quality/benchmarking metrics or service goals). | There is a perceived need to reduce the pressure from contractual time constraints. YTYAIC may offer a way to do this (how YTYAIC is implemented may be a moderating factor). | '*Well, I mean, it's a balance, because the extra time, you know, you can very easily argue that the extra time we're spending doing, you know, the specific CBT or going through the booklet is going to save us a hell of a lot of time in terms of getting, you know, cooperation from the patient. I mean, that in itself is very, very time consuming. So, I think from a time point of view, if, you know, if it's done in the right way with the right patients, it potentially could save a lot of time*.’ (S17, principal dentist, male, intervention) |

Potential Barriers

| **Innovation Domain** | | | |
| --- | --- | --- | --- |
| **CFIR Construct** | **Definition** | **Potential Barrier** | **Quote(s)** |
| Evidence/ Relative advantage | The innovation has robust evidence supporting its effectiveness.  The innovation is better than other available innovations or current practice | The future use of YTYAIC may depend on quantitative evidence of its effectiveness. CALM may not result in such evidence. | *‘It's very easy for clinicians to view this as a bit of a soft science thing* [...] *like it's not as tangible as saying, “oh, the statistics of this works this way and this works that way”*’ (S01, foundation dentist, female, intervention)  '*I'd be interested in what the results are as to whether using the CBT would be helpful. If it is, then that’s something I'd be interested in doing*.’ (S04, principal dentist, female, not allocated within CALM) |
| Evidence/ Relative advantage | The innovation has robust evidence supporting its effectiveness.  The innovation is better than other available innovations or current practice | Caregivers may question the effectiveness of a self-guided CBT approach. | *‘They asked me something like “but does that even work?”*’ (S01, foundation dentist, female, intervention) |
| Cost | The innovation purchase and operating costs are affordable. | The ongoing cost of purchasing copies of YTYAIC reduces its potential future use. | ‘*The cost wouldn’t be huge in terms of the number of books that we would want but it’s an added cost onto the contract that’s already stretched.* […] *I certainly wouldn’t be giving it to every child if it was costing us.*’ (S13, principal dentist, female, intervention) |
| **Implementation Process Domain** | | | |
| **CFIR Construct** | **Definition** | **Potential Barrier** | **Quote(s)** |
| Teaming | Join together, intentionally coordinating and collaborating on interdependent tasks, to implement the innovation. | Other members of staff may undermine implementation by providing messages that conflict with YTYAIC. | *‘Your nurse may say something like, “oh, it's not that bad”, you know, or something like—do you know what I mean, something where, then for you to turn around and introduce this resource like, “oh, but you get to control what happens here”, it just kind of doesn't, everybody's not on the same page.*’ (S01, foundation dentist, female, intervention) |
| Assessing Needs | Collect information about priorities, preferences, and needs of people to guide implementation and delivery of the innovation. | Dental professionals’ assessment of treatment needs or level of anxiety results in decisions not to use YTYIAC. | ‘*If they need like multiple treatments like lots of treatment and lots of difficult treatment then it's sort of weighing up like maybe we could get a bit of work done using it but is it actually better if they have some like sedation or a GA and got it all done, like is it too much to go through*.’ (S04, principal dentist, female, not allocated within CALM) |
| Engaging and Doing | Attract and encourage participation in implementation and/or the innovation. | Dental professionals may not spend time and fully engage with YTYAIC when introducing the resource to patients (motivation of dental professionals may be a moderating factor) | *‘But some of the dentists wouldn’t spend time going through it, they just give it to the patient*.’ (S18, foundation dentist, female, intervention) |
| Reflecting and Evaluating | Collect and discuss quantitative and qualitative information about the success of implementation and intervention. | Evaluation of negative patient responses related to the way YTYAIC is implemented. | ‘*That's the only thing that I would say is a bit tricky, there's a lot of reading and there's a lot to go through in the booklet. And sometimes, patients just look at you thinking, “what's going on here, how long is he going to talk for about this?”* (S12, principal dentist, male, intervention) [dentist who describes going through YTYAIC ‘*page by page’*] |
| **Individual Domain** | | | |
| **CFIR Construct** | **Definition** | **Potential Barrier** | **Quote(s)** |
| Need | The individual(s) has deficits related to survival, well-being, or personal fulfilment, which will be addressed by implementation and/or delivery of the innovation. | Higher levels of anxiety may be perceived as being related to patients rejecting YTYAIC. | *‘I guess it’s sort of proportionate to their anxiety as well. If they're more anxious, they don't tend to fill it in as well. Which I think is just more of a sort of, they just reject it more, I don't know.*’ (S09, associate dentist, male, intervention) |
| Capability | The individual(s) has interpersonal competence, knowledge, and skills to fulfil role. | Dental professionals with less experience treating children and engaging with caregivers may find it more difficult to use YTYAIC. | ‘*I think if you are someone that doesn't treat children very often, that might be a bit more difficult and you have to be confident in getting the parent on board as well because I think that's a big part of it*.’ (S02, associate dentist, female, intervention) |
| Capability | The individual(s) has interpersonal competence, knowledge, and skills to fulfil role. | Literacy levels among patients and/or caregivers may impact on the use of YTYAIC. | ‘*One to be fair was quite – I don’t know how to describe them really, a socially deprived family and they certainly had probably some learning difficulties although mild, but as a family as a whole. So, I don’t think they particularly read or did anything. So, although they could read, it was a struggle for them and things like that.’* (S13, principal dentist, female, intervention) |
| Capability | The individual(s) has interpersonal competence, knowledge, and skills to fulfil role. | Patients’ ability to address the questions in YTYAIC. | ‘*But when I actually like had it to do I found it harder to explain; I didn’t want to like not make it clear. And like I wanted to say how I felt, but I didn’t know how to write it*.’ (C01, patient aged 11, female) |
| Opportunity | The individual(s) has availability, scope, and power to fulfil role. | Dental professionals may lack the time to use YTYAIC. This depends on the constructs Inner Setting – Delivery – Available Resources and Outer Setting – External Pressure. | *‘All of the interventions within dentistry, the issue comes when you try to implement it and no one’s got any extra time, no one’s got a spare minute in their check-ups and no one’s got any spare funding to have anyone else do it.*’ (S16, practice manager, female, not allocated within CALM) |
| Opportunity | The individual(s) has availability, scope, and power to fulfil role. | Patients and/or caregivers do not have the time to complete YTYAIC at home. | *‘They weren't from the, they had kind of busy family lives that then trying to ask that to be filled out at home was just a no-go. I tried it the first time and then I just thought “it's not going to happen”.* (S15, community dentist, female, intervention) |
| Motivation | The individual(s) is committed to fulfilling role. | Dentists may lack the motivation to help patients to manage their anxious thoughts, feelings, behaviour and physical symptoms | ‘*Whereas perhaps some dentists, they're more keen on getting the treatment done, they enjoy doing the filling, they enjoy that part. If the patient is anxious on the end of that, you know, if the patient's anxious receiving that, they don't respond well to that. You know, they don't want to do that*.’ (S10, community dentist, female, usual care)  ‘*My worry would be if it's not, if you have to go through all them parts, and just doing it half-heartedly or half of it might not be enough*.’ (S12, principal dentist, male, intervention) |
| Motivation | The individual(s) is committed to fulfilling role. | Dentists may lack the motivation to spend time learning about YTYAIC. | ‘*I think with anything like this, it's the barrier to understanding, so you’d have to take some time out of either your personal life or your work life to really understand the resource and how to use it and why it works. And I think that would automatically create a bit of a threshold for people who don't buy into it from the immediate outset are very unlikely to then take time to learn it*.’ (S01, foundation dentist, female, intervention) |
| Motivation | The individual(s) is committed to fulfilling role. | Patients may not engage with YTYAIC at home. | ‘*I don't think that they're quite doing it at home and they’re definitely not bringing it back.*' (S05, dental therapist, female, intervention) |
| Motivation | The individual(s) is committed to fulfilling role. | Older children may be less motivated to engage with YTYAIC. | ‘*One, was a kind of teenager, I don't know, about 14 years old or something like that and she was kind of getting almost bored with the resources and going through them. And like even at the first visit, I tried to kind of discuss things and she just said, “okay, I'm bored now, just get on with it*”.’ (S15, community dentist, female, intervention) |
| Motivation | The individual(s) is committed to fulfilling role. | Caregivers may not be motivated to support the child to use YTYAIC and complete the Message to Dentist. | ‘*But if parents aren’t that helpful and the patient’s confused then you can sort of get sort of stuck in a rut with it where [.] when they come back next time, they haven’t filled it out properly and they still they can't understand what is happening.*’ (S09, associate dentist, male, intervention) |
| **Inner Setting Domain** | | | |
| **CFIR Construct** | **Definition** | **Potential Barrier** | **Quote(s)** |
| Delivery – Available Resources | Resources are available to implement and deliver the innovation. | Potential for YTYAIC to require additional resources in terms of time. | ‘*The only thing I would say is I don't know how much time it would take. And time is the big thing, certainly within NHS, busy NHS practices. So, the impact it would have would be on resources of time more than anything and whether we could fit in the CBT intervention into our routine appointments, or would it require extra time, and extra burden.*’ (S08, principal dentist, male, usual care) |
| **Outer Setting Domain** | | | |
| **CFIR Construct** | **Definition** | **Potential Barrier** | **Quote(s)** |
| External Pressure | External pressures drive implementation and/or delivery of the innovation (e.g. mass media campaigns, competing with peer entities. Quality/benchmarking metrics or service goals). | Lack of time within contractual arrangements to spend time on managing anxiety. | ‘*Yeah, that would be if you were to go into NHS dentistry, that's the sort of speed you’d be expected to work at* [had previously mentioned 15 or sometimes 10-minute exam appointments]. *So, for my colleagues, children with dental anxiety, even though you could probably coach them into getting used to the environment, they just don't necessarily have the time to do that really*.’ (S01, foundation dentist, female, intervention) |
